# Morphometric Analysis of Pedicles of Typical Thoracic Vertebrae for Transpedicular Screw Fixation: A Study From Eastern India

**DOI:** 10.7759/cureus.114761

**Published:** 2026-08-18

**Authors:** Gyanaranjan Nayak, Sujita Pradhan, Lopamudra Nayak, Niranjan Sahoo, Saswati Mohanty, Pravash Parida, Gyanraj Singh, Saurjya R Das

**Affiliations:** 1 Anatomy, Institute of Medical Sciences (IMS) and SUM Hospital, Campus II, Siksha 'O' Anusandhan Deemed to be University, Bhubaneswar, IND; 2 Forensic Medicine, All India Institute of Medical Sciences (AIIMS), Bhopal, Bhopal, IND; 3 Orthopaedics, Institute of Medical Sciences (IMS) and SUM Hospital, Campus II, Siksha 'O' Anusandhan Deemed to be University, Bhubaneswar, IND; 4 Anatomy, Dharanidhar Medical College and Hospital, Keonjhar, IND

**Keywords:** chord length, pedicle angle, pedicle morphometry, prosthesis, transpedicular screw fixation, typical thoracic vertebra

## Abstract

Introduction: Transpedicular screw fixation has emerged as an indispensable technique in thoracic spine surgery for conditions ranging from traumatic fractures to deformity correction and tumour resection. Precise morphometric knowledge of pedicle dimensions is mandatory to avoid neurovascular complications during screw placement. Data from the Indian subcontinent, and particularly from Eastern India, remain sparse, underscoring the need for region-specific anatomical studies.

Methods: A descriptive morphometric study was conducted at the Institute of Medical Sciences and SUM Hospital Campus II, Bhubaneswar, Odisha, between January 2024 and March 2026. Pedicle height (PH), pedicle width (PW), chord length (CL), interpedicular distance (IPD), transverse pedicle angle (TPA), and sagittal pedicle angle (SPA) were measured bilaterally on 106 specimens of typical thoracic vertebrae (T2-T8) using Vernier callipers and a goniometer as per standardised protocols.

Results: Mean PH was 10.56 ± 1.24 mm (right) and 10.56 ± 1.31 mm (left). Mean PW was 4.93 ± 0.96 mm (right) and 5.02 ± 0.94 mm (left). Mean CL was 37.06 ± 4.84 mm (right) and 38.14 ± 4.44 mm (left). Mean IPD was 14.78 ± 1.50 mm. Mean TPA was 12.57 ± 1.78° (right) and 12.49 ± 1.17° (left). Mean SPA was 13.60 ± 1.50° (right) and 10.78 ± 0.76° (left).

Conclusion: The pedicle dimensions recorded in this Eastern Indian cohort are distinct from those reported in Western and other Asian populations. The narrow pedicle width observed at mid-thoracic levels warrants cautious screw selection. These data provide a clinically relevant morphometric reference to guide safer transpedicular instrumentation in the region.

## Introduction

Pedicle screw fixation is presently regarded as the gold standard for posterior instrumentation of the thoracic spine. Vaccaro et al. [1] reported that the technique allows three-column spinal stabilisation, facilitating effective correction of deformity, stabilisation of fractures, and tumour resection with acceptable complication profiles when executed meticulously. Transpedicular screw fixation of the thoracic spine poses considerable surgical challenges owing to its small cross-sectional dimensions, particularly in the mid-thoracic region (T4-T8), and its intimate spatial relationship with the spinal cord, nerve roots, and great vessels. Intraoperative pedicle screw misplacement rates in the thoracic spine have been reported to range from 3% to as high as 55% in various series, with medial wall breaches carrying the most serious sequelae, including spinal cord injury [2,3]. The dimensions that surgeons must appreciate include pedicle width (the narrowest transverse diameter), pedicle height (the sagittal diameter at the isthmus), the transverse pedicle angle (TPA) dictating medial angulation, the sagittal pedicle angle (SPA) governing cranial or caudal trajectory, chord length (representing screw path length from the posterior laminar cortex to the anterior vertebral body cortex), and interpedicular distance [4].

Studies from other countries have reported that the thoracic spinal dimensions of Indians differ considerably from those of China, Japan, South Korea, and Paris [5-7].

Eastern India, encompassing states such as Odisha, West Bengal, Jharkhand, and Bihar, harbours a large tribal and indigenous population with anthropometric features that may differ from metropolitan Indian cohorts. Despite the region harbouring numerous tertiary spine surgery centres, robust morphometric data from this geography are conspicuously lacking. Surgeons in this region continue to rely on reference data derived from other populations, which may predispose to implant mismatch, inadequate fixation, or inadvertent neurovascular injury.

The present descriptive study was therefore designed to measure six critical pedicle morphometric parameters, pedicle width, pedicle height, chord length, interpedicular distance, transverse pedicle angle, and sagittal pedicle angle, bilaterally in 106 typical thoracic vertebrae from a tertiary care centre in Bhubaneswar, Odisha, and to compare these findings with existing literature, with the ultimate aim of generating region-specific normative data to inform transpedicular screw selection and trajectory planning in Eastern India.

## Materials and methods

Study design and setting

This is a descriptive and cross-sectional morphometric study conducted in the Department of Anatomy, IMS and SUM Hospital Campus II, Phulnakhara, Siksha 'O' Anusandhan (Deemed to be University), Bhubaneswar, Odisha, India. Data were collected from January 2024 to March 2026.

Sample selection

A total of 106 dried typical thoracic vertebrae (T2 through T8) were included. Vertebrae were drawn from the institutional osteological collection. Specimens were included if they were skeletally mature, well-preserved, and intact without fractures, deformities, or evidence of pathological lesions. Vertebrae with damaged or broken pedicles, gross structural deformity, or incomplete ossification were excluded. The study was approved by the Institutional Ethics Committee, IMS and SUM Hospital Campus II, Siksha 'O' Anusandhan (Deemed to be University), Bhubaneswar, Odisha (Letter No. IEC/IMS2/2026/07/35; July 22, 2026).

Parameter definitions

**Figure 1:**
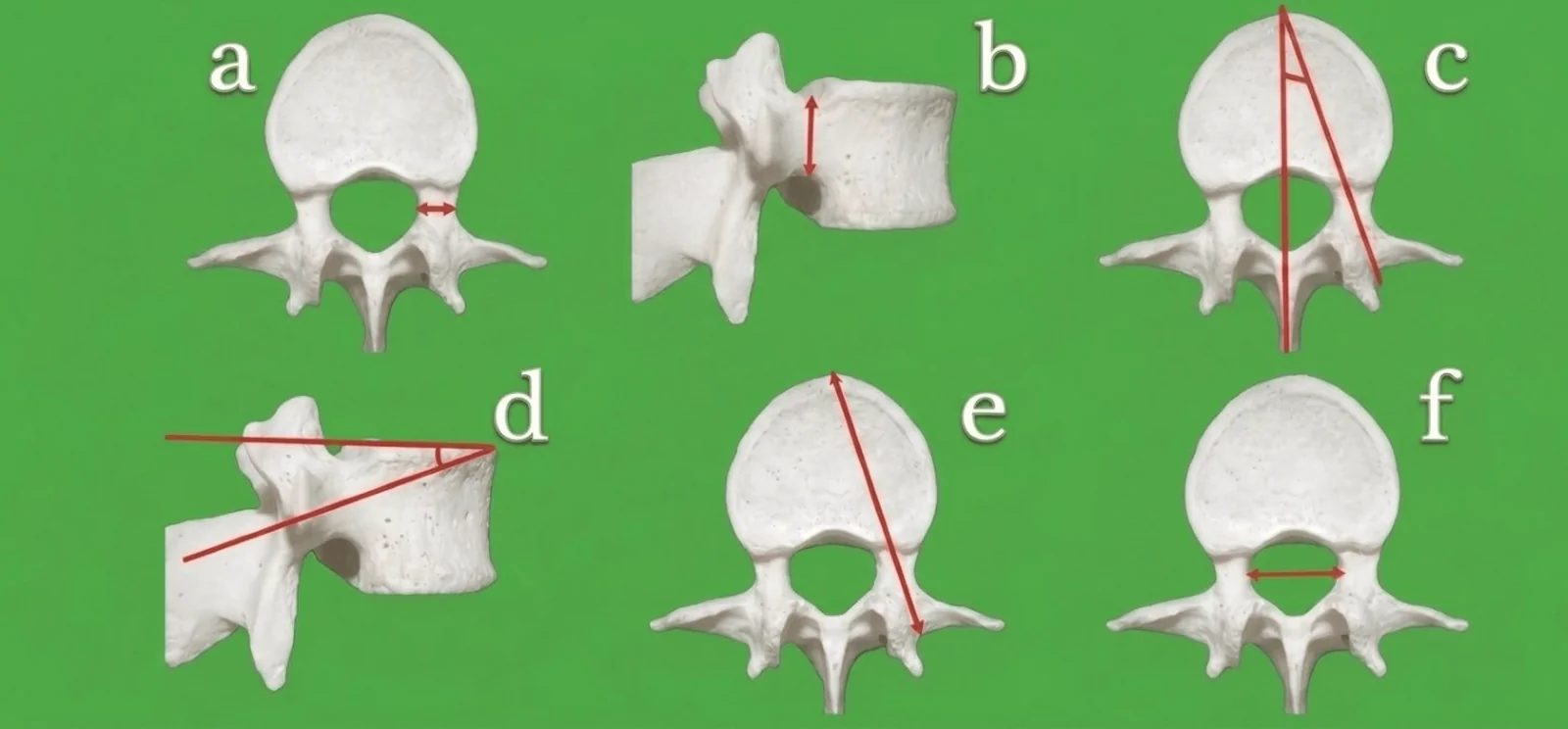
Photographs Showing the Various Measurements Taken on the Vertebra (a) Pedicle width, (b) pedicle height, (c) transverse angle, (d) sagittal angle, (e) chord length, and (f) interpedicular distance.

Mwakikunga et al. outlined six morphometric parameters measured bilaterally on each vertebra using conventional techniques (Figure 1) [4].

Pedicle Width (PW)

The transverse diameter at the isthmus of the pedicle. This was measured at the midpoint of the pedicle, perpendicular to its long axis, between the medial and lateral surfaces. A line perpendicular to and bisecting the pedicle's smallest diameter was designated as the pedicle axis. A Vernier calliper was used for the measurement.

Pedicle Height (PH)

A Vernier calliper was used to measure the sagittal diameter at the pedicle isthmus, which is the vertical distance between the pedicle's superior and inferior edges at its midpoint.

Transverse Pedicle Angle (TPA)

The angle was measured with a goniometer between the mid-sagittal plane and the plane that bisects the pedicle in the transverse direction.

Sagittal Pedicle Angle (SPA)

A goniometer was used to measure the angle formed by a line that passes across the pedicle axis and the superior vertebral border in the sagittal plane.

Chord Length (CL)

A Vernier calliper was used to measure the distance along the pedicle axis between the anterior cortex of the vertebral body and the most posterior portion of the lamina-cortex junction. This is the ideal length for the screw path.

Interpedicular Distance (IPD)

Using Vernier callipers, the separation between the medial surfaces of the right and left vertebral pedicles was measured at the isthmus level.

Measurement protocol

To reduce inter-observer variability, only one qualified anatomist carried out each measurement. Every parameter was measured three times, and the average result was noted. Standardised lighting conditions were used for the measurements. For every recording, a standard stainless-steel goniometer with a graduation of 1° and a Mitutoyo digital Vernier calliper with an accuracy of 0.01 mm were used.

Statistical analysis

IBM SPSS Statistics for Windows, Version 26 (Released 2018; IBM Corp., Armonk, New York) was used to analyse the data after entering the data into Microsoft Excel (Microsoft Corporation, Redmond, Washington). For every parameter, descriptive statistics such as mean, standard deviation (SD), minimum, and maximum were calculated. The paired t-test was used to evaluate bilateral symmetry. Statistical significance was defined as a p-value of less than 0.05.

## Results

A total of 106 typical thoracic vertebrae were studied. The morphometric data for all six parameters are summarised in Tables 1-3. Pedicle height and pedicle width are presented in Table 1. The mean PH was 10.56 ± 1.24 mm on the right and 10.56 ± 1.31 mm on the left, with no statistically significant bilateral difference (p > 0.05). PH ranged from 7.79 to 13.51 mm on the right and 7.68 to 13.58 mm on the left. The mean PW was 4.93 ± 0.96 mm on the right and 5.02 ± 0.94 mm on the left. The narrowest pedicle widths (<4.0 mm) were encountered in specimens corresponding to the mid-thoracic levels (T4-T8), consistent with the well-documented hourglass narrowing at these levels. No statistically significant bilateral asymmetry was identified (p > 0.05).

**Table 1:** Pedicle Height and Pedicle Width (mm): Descriptive Statistics

Parameter	Side	n	Mean ± SD	Minimum	Maximum
Pedicle height (mm)	Right	106	10.56 ± 1.24	7.79	13.51
Pedicle height (mm)	Left	106	10.56 ± 1.31	7.68	13.58
Pedicle width (mm)	Right	106	4.93 ± 0.96	3.03	7.33
Pedicle width (mm)	Left	106	5.02 ± 0.94	3.06	7.39

Chord length and interpedicular distance are presented in Table 2. The mean CL was 37.06 ± 4.84 mm (right) and 38.14 ± 4.44 mm (left). CL ranged from 14.12 to 47.08 mm on the right and from 24.90 to 47.84 mm on the left. The chord length generally increased from upper thoracic to lower thoracic vertebrae, reflecting increasing vertebral body depth. The mean IPD was 14.78 ± 1.50 mm, ranging from 11.06 to 18.59 mm. The smallest IPD values observed were below 12 mm, corresponding to mid-thoracic specimens, and merit attention when planning bilateral transpedicular instrumentation at those levels.

**Table 2:** Chord Length (mm) and Interpedicular Distance (mm): Descriptive Statistics

Parameter	Side	n	Mean ± SD	Minimum	Maximum
Chord length (mm)	Right	106	37.06 ± 4.84	14.12	47.08
Chord length (mm)	Left	106	38.14 ± 4.44	24.90	47.84
Interpedicular distance (mm)	Bilateral	106	14.78 ± 1.50	11.06	18.59

Pedicle angles (transverse and sagittal) are presented in Table 3. The mean TPA was 12.57 ± 1.78° (right) and 12.49 ± 1.17° (left), indicating that screws should be directed medially at approximately 12-13° from the mid-sagittal plane. The mean SPA was 13.60 ± 1.50° (right) and 10.78 ± 0.76° (left). A statistically significant bilateral asymmetry was noted for SPA (p < 0.05), with the right side exhibiting a consistently larger cephalad angulation.

**Table 3:** Transverse Pedicle Angle and Sagittal Pedicle Angle (Degrees): Descriptive Statistics

Parameter	Side	n	Mean ± SD	Minimum	Maximum
Transverse pedicle angle (°)	Right	106	12.57 ± 1.78	7	18
Transverse pedicle angle (°)	Left	106	12.49 ± 1.17	10	17
Sagittal pedicle angle (°)	Right	106	13.60 ± 1.50	10	17
Sagittal pedicle angle (°)	Left	106	10.78 ± 0.76	10	13

## Discussion

This descriptive morphometric investigation presents, to the best of our knowledge, one of the few comprehensive quantifications of pedicle parameters in typical thoracic vertebrae derived specifically from an Eastern Indian tertiary care institution. The findings have direct clinical relevance for spine surgeons operating in this region, where the patient population exhibits distinct somatic characteristics compared with Western and East Asian cohorts.

The mean PW of 4.93 mm (right) and 5.02 mm (left) recorded in our series is broadly aligned with several other Indian series but differs considerably from that in Caucasians (6-8 mm at the mid-thoracic level) [8]. Singh et al. [9] and Datir et al. [10] have reported similar values of mean PW in the Indian population. The narrow mid-thoracic pedicles observed in our study underscore the limited margin for screw diameter selection. In such cases, surgeons should consider smaller-diameter (3.5-4.0 mm) screws to avert medial wall perforation.

The mean PH of 10.56 mm bilaterally is of practical significance because the height of the pedicle is less restrictive than the width in determining screw diameter, and a relatively preserved PH at mid-thoracic levels allows confident craniocaudal trajectory adjustment. Several Asian studies, including Xu et al. [5] and Hou et al. [11] from China, have similarly shown that PH is consistently larger than PW throughout the thoracic spine, with PH exceeding 10 mm from T1 to T10.

The mean CL of 37.06 mm (right) and 38.14 mm (left) in our study provides a useful intraoperative reference for screw length selection. Screw penetration to 80% of the chord length is the conventionally recommended target to achieve maximal pullout strength without risking anterior cortical breach and great vessel injury. Based on our data, a screw length of 28-30 mm at upper thoracic levels and 35-40 mm at lower thoracic levels would be appropriate for the majority of Eastern Indian patients. The minimum CL observed (14.12 mm, right side) is likely attributable to an upper thoracic (T1-T2) specimen, where shorter screw paths are expected, and the surgeon must exercise particular vigilance to avoid anterior perforation [12,13].

The mean TPA of approximately 12.5° bilaterally is compatible with data from Asian studies [14,15] and corresponds to the medial inclination required for safe screw passage through the pedicle canal.

The mean SPA of 13.60° (right) and 10.78° (left) suggests that a cephalad inclination of approximately 10-14° is appropriate for screw trajectory in the sagittal plane, with a significant right-left asymmetry in SPA noted in our series. This asymmetric finding may relate to asymmetric costovertebral articulation or minor morphological lateralisation intrinsic to the Eastern Indian skeletal phenotype; further investigation is warranted.

When contrasted with global data, our findings show that the Eastern Indian thoracic pedicle is narrower (PW ~4.9-5.0 mm) but of comparable height to those in published Chinese, Korean, and Turkish series [5,6,11,16,17]. The mean TPA and SPA in our study are consistent with data from studies conducted in China [5] and Turkey [18] but diverge from larger-framed Caucasian series where TPA tends to be slightly higher at upper thoracic levels [19].

The present study is strengthened by its exclusive focus on typical thoracic vertebrae (T2-T8), ensuring applicability to the clinical scenarios most commonly requiring thoracic instrumentation, a relatively large sample of 106 specimens, and standardised measurement methodology employing a single trained observer. Limitations include the retrospective design, the use of dried bone specimens (which may exhibit minor shrinkage artefact), absence of stratification by vertebral level (which precludes level-by-level analysis), and lack of sex-disaggregated data owing to the unavailability of reliable skeletal sex markers for all specimens. Future studies should incorporate computed tomographic morphometry, include sex-specific and level-specific analyses, and correlate morphometric data with clinical outcomes of pedicle screw fixation in this population [20].

## Conclusions

The morphometric analysis of 106 typical thoracic vertebrae from the Eastern Indian cohort reveals that pedicle width is the most constrained dimension, particularly at mid-thoracic levels, where mean PW falls below 5 mm. Chord length, interpedicular distance, and pedicle angles were comparable to other Asian series but consistently smaller than Caucasian reference data. A significant asymmetry in sagittal pedicle angle between the right and left sides was identified and warrants further investigation. These normative data from Odisha provide an anatomically accurate, population-specific foundation for pre-operative planning of transpedicular screw fixation in Eastern Indian patients, potentially reducing implant mismatch and iatrogenic neurovascular complications.
